# COVID-19 and Cardiomyopathy in African Americans: An Early Single-Center Experience

**DOI:** 10.7759/cureus.38529

**Published:** 2023-05-04

**Authors:** Ammar Ahmed, Andrew D Assaf, Navid Khamooshi, Grace D Brannan, Souheil Saba, Marcel E Zughaib

**Affiliations:** 1 Department of Cardiovascular Medicine, Ascension Providence Hospital, Southfield, USA; 2 Department of Internal Medicine, Ascension Providence Hospital, Southfield, USA; 3 Medical Education, GDB Research and Statistical Consulting, Athens, USA

**Keywords:** non-ischemic cardiac pathologies, transthoracic echocardiogram, african american/black, covid 19, congestive heart faiulre

## Abstract

Introduction

The 2019 coronavirus pandemic has taken a toll on our society. Although most patients report minimal symptoms, a small proportion of patients have reported significant respiratory symptoms that led to admission to the inpatient medical ward or even the intensive care unit. Complications and long-term sequela of COVID-19 are still being reported and studied. The presence of cardiomyopathy, whether established or new-onset and its effect on inpatient mortality, admission to the intensive care unit or length of stay hasn’t been studied.

Methods

All inpatient hospitalizations in our database between March 1, 2020, and April 30, 2020, due to COVID-19 were reviewed. Patients who had at least a limited echocardiogram during this time were included in the study if they were above the age of 18. Patients were then assigned to three groups. The first group had patients with normal left ventricular systolic function. The second group had established cardiomyopathy that persisted throughout admission. The third group had patients who were found to have new-onset cardiomyopathy during admission.

Results

The inpatient mortality, although high and variable, wasn’t significantly different between the three groups. Also, there was no significant difference between admission to the intensive care unit, disposition at discharge, or oxygenation status at 24 hours between the three groups. The length of stay in the established cardiomyopathy group was markedly lower, and we suspect that could be due to more aggressive discussions about end-of-life care.

Conclusion

Early COVID-19 experience at our center revealed a relatively high mortality rate that was primarily due to respiratory failure. The presence of established or new cardiomyopathy didn’t appear to alter the outcomes significantly early in the pandemic.

## Introduction

Coronaviruses are a part of the Coronaviridae family and primarily cause respiratory infections in humans and other mammalian species [1]. Coronavirus infection in humans may be silent or accompanied by fever, rhinorrhea, shortness of breath, and gastrointestinal symptoms [1]. In rare scenarios, particularly in immunocompromised patients, coronavirus infection may lead to respiratory failure, multi-organ dysfunction and subsequent death [1]. To this date, three major outbreaks attributed to coronavirus infection have been reported worldwide: The first started off as an outbreak in a southern province of China in 2002, which was later on known as severe acute respiratory distress syndrome or SARS. Eight thousand ninety-eight infections were attributed to SARS in 26 countries resulting in a death toll of 774 individuals in a span of one year [2]. Ten years later, in 2012, a similar outbreak occurred in the Middle East caused by another coronavirus strain, which was later on termed Middle East Respiratory Syndrome or MERS [2]. More than 2500 infections attributed to MERS were reported in more than 27 countries, with a death toll of around 866 [2]. The third and most impressive outbreak of another novel coronavirus started off in Wuhan, China, in late 2019, which has resulted in the current 2019-nCoV global pandemic that we have been battling to this date [2]. Following the rapid spread of this particular viral strain, the World Health Organization (WHO) declared a global emergency on January 30, 2020 [2]. Based on the Johns Hopkins coronavirus resource center, as of January 27, 2022, the current 2019-nCoV global pandemic has resulted in more than 365 million infections and 5.5 million deaths globally, of which more than 73 million infections and at least 850,000 deaths occurred in the United States alone [3]. Almost 10 billion vaccination doses against this deadly virus have been given worldwide [3].

Multiple complications have been reported in patients with the 2019-nCoV infection. They are more commonly seen in patients with severe disease and include cytokine release syndrome, acute respiratory distress syndrome, arrhythmia, heart failure, myocarditis, venous thromboembolism and pulmonary embolism, stroke, encephalopathy, and acute kidney injury, in addition to secondary infections [4]. Other long-term complications are being reported and studied currently. 

Of particular interest, the risk of myocarditis in the setting of the current 2019-nCoV infection is higher than in the general population [5]. Specifically, the CDC has reported a 42.3% increase in inpatient encounters due to myocarditis between the years 2019 and 2020 [5]. In one study involving more than 900 hospitals, patients infected with the 2019-nCoV were almost 16 times more likely to have myocarditis than patients admitted without the infection, although the overall incidence was low in both groups (0.146% vs. 0.009%, respectively) [5]. Proposed mechanisms of myocarditis include direct cardiomyocyte injury, pericardial infection, ischemia secondary to microvascular disease, and proinflammatory cytokines resulting in arrhythmias [6]. The clinical presentation of coronavirus-mediated myocarditis may range from mild symptoms such as mild dyspnea and fatigue to profound tachycardia, unstable arrhythmias, and left ventricular systolic dysfunction with cardiogenic shock within a few weeks of contracting 2019-nCoV [6]. Differential diagnoses of presumed coronavirus-related myocarditis include acute coronary syndrome, sepsis-related cardiomyopathy, and stress-induced cardiomyopathy (Takotsubo cardiomyopathy) [6]. Diagnostic considerations include obtaining cardiac biomarkers (i.e., troponin and pro-BNP), electrocardiogram (ECG), imaging studies such as an echocardiogram, coronary computed tomography angiography (CCTA) or cardiac magnetic resonance imaging (CMR), and rarely endomyocardial biopsy (EMB) [6]. Management involves treating the underlying infection along with managing complications of myocarditis [6]. 

Our study aimed to determine the outcomes of patients admitted to our hospital between March 1, 2020, and April 30, 2020, who were diagnosed with the 2019-nCoV and also had established cardiomyopathy or new-onset cardiomyopathy based on history and a limited echocardiogram.

## Materials and methods

Study design

All patients who were admitted to our hospital system, which included two sites (Ascension Providence Hospital in Southfield, MI, and Ascension Providence Park Hospital in Novi, MI), between the dates of March 1, 2020 and April 30, 2020, with COVID-19 were screened. Our intention was to place patients, retrospectively, in three different groups. The first group being patients who were admitted to the hospital with a primary diagnosis of COVID-19 and who had normal left ventricular systolic function at baseline defined by an ejection fraction of ≥50% by transthoracic echocardiography. This group served as our control group. The second group included patients with an established history of cardiomyopathy defined by documentation of a left ventricular ejection fraction of <50% on a prior echocardiogram who were admitted with a primary diagnosis of COVID-19. The third group included patients with no history of cardiomyopathy who were admitted with a primary diagnosis of COVID-19 and were found to have new-onset left ventricular dysfunction (ejection fraction <50%). The study was reviewed and approved by the Institutional Review Board of the Ascension Providence/Providence Park Hospital system. The primary endpoints include mortality rate, admission to the intensive care unit, length of stay, disposition at discharge, oxygenation status within 24 hours of admission, and cardiac biomarkers and markers of acute inflammation. A subgroup analysis based on ejection fraction was also performed.

Inclusion and exclusion criteria

**Figure 1:**
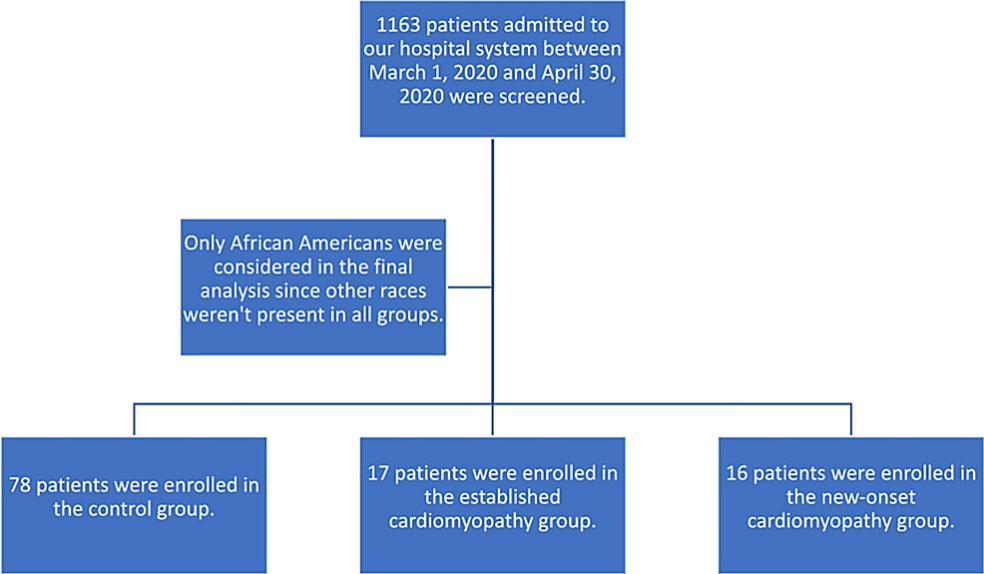
One thousand one hundred sixty-three patients were screened for eligibility. Eighty-five total patients had echocardiograms demonstrating normal left ventricular systolic function. Eighteen patients qualified for the established cardiomyopathy group, and 17 patients qualified for the new-onset cardiomyopathy group. After the exclusion of races other than African American, the final numbers were 78, 17, and 16 for the control group, the established cardiomyopathy group, and the new-onset cardiomyopathy group, respectively.

All eligible patients had to be at least 18 years old and had to be admitted with a primary diagnosis of COVID-19. Having an echocardiogram during the same hospitalization demonstrating normal left ventricular systolic function was a requirement for patient enrollment in the control group. The electronic medical record was used for data review and collection (Figure 1).

Variables

The electronic medical record was used for data review and collection. Baseline characteristics (age, sex, gender, etc.) were obtained. Furthermore, various medical comorbidities were noted (e.g., coronary artery disease, chronic kidney disease, systemic hypertension, hyperlipidemia, etc.). The goal of this study was to compare outcomes between the three groups (mortality rate, outcomes at discharge, length of stay, admission to the intensive care unit, oxygenation status, cardiac biomarkers, and markers of acute illness). 

Statistical analysis

For continuous variables, groups were compared using an analysis of variance (ANOVA) across three groups and an independent t-test between two groups. A post-hoc analysis was done to determine if the difference, if found, is significant. Least significant difference (LSD) was computed to separate means across the three groups for variables found to be significant. A Pearson chi-square test was performed to compare groups for categorical variables. For continuous variables, values were expressed as mean (±SD), whereas for categorical variables, values were expressed as n (%). Data analyses were performed using IBM SPSS Statistics for Windows, Version 28.0 (IBM Corp., Armonk, NY). Significance was set at a p-value of 0.05. Also, races other than African American were removed because of the small sample size and because they were only found in the control or established groups and not the new-onset group.

## Results

A total of 1163 patients were admitted to our hospital system (Ascension Providence and Ascension Providence Park Hospitals) between March 1, 2020, and April 30, 2020. After applying the inclusion criteria, 85 patients qualified for the control group, 18 for the established cardiomyopathy group, and 17 for the new-onset cardiomyopathy group. Races other than African American were not present in all groups, and, therefore, only African American patients were included in the final analysis. After the exclusion of races other than African American, the final numbers were 78, 17, and 16 for the control group, the established cardiomyopathy group, and the new-onset cardiomyopathy group, respectively.

Baseline characteristics, including age, body mass index, sex, history of smoking, and alcohol use, in addition to various laboratory results, are listed in Table 1. The mean age was approximately 64.9 ± 14.8, 68.8 ± 13.5, and 63.3 ± 10.6 in the control, established cardiomyopathy, and new-onset cardiomyopathy groups, respectively (p-value NS). The average BMI was approximately 34.2 ± 9, 28.9 ± 6.4, and 32.1 ± 6.4 kg/m^2^ in the control, established cardiomyopathy, and new-onset cardiomyopathy groups, respectively (p-value NS). The mean ejection fraction was approximately 60.6% in the control group, 29.2% in the established cardiography group, and 31.3% in the new-onset cardiomyopathy group. The highest percentage of male patients was in the established cardiomyopathy group (thirteen of seventeen patients-76.5%), while the highest percentage of smokers was in the new-onset cardiomyopathy group (three of sixteen patients-18.75%). The highest creatinine level was present in the new-onset cardiomyopathy group, with an average of 4.47 (p-value 0.035).

**Table 1:** Baseline characteristics (average values). *Statistically significant. Values were expressed as mean (±SD), whereas for categorical variables, values were expressed as n (%).

	Control (N = 78)	Established CM (N = 17)	New-onset CM (N = 16)	P-value
Age (years)	64.9 ± 14.79	68.76 ± 13.53	63.31± 10.56	0.497
Body Mass Index (Kg/m^2^)	34.17 ± 8.97	28.86 ± 6.35	32.13 ± 6.39	0.059
Sex (male)	33.0 (42.3%)	13 (76.5%)	10.0 (62.5%)	0.033*
Smoking (current)	3.0 (3.85%)	2 (11.76%)	3 (18.75%)	0.091
Smoking (past)	18.0 (23.10%)	7 (41.18%)	2 (12.5%)	
Alcohol (current)	8.0 (10.3%)	0	0	0.158
Alcohol (past)	5.0 (6.4%)	2.0 (12.5%)	1.0 (6.3%)	
Race				
African Americans only	78	17	16	
White blood cell count (1000/µL)	7.71 ± 4.99	8.08 ± 3.07	8.82 ± 3.55	0.672
Hemoglobin (g/dl)	12.39 ± 2.25	11.81 ± 2.71	11.61 ± 2.54	0.376
Platelets (1000/µL)	199.91 ± 75.47	207.94 ± 97.90	230.00 ± 94.16	0.408
Prothrombin time (s)	14.69 ± 3.25	16.75 ± 5.20	18.65 ± 18.55	0.285
International normalized units	1.26 ± 0.278	1.43 ± 0.439	1.60 ± 1.59	0.284
Blood urea nitrogen (mg/dl)	28.19 ± 20.40	42.29 ± 18.68	34.87 ± 19.13	0.027*
Creatinine (mg/dl)	2.17 ± 2.86	2.72 ± 2.23	4.47 ± 5.11	0.035*
Na (mmol/L)	138.26 ± 4.81	138.19 ± 3.45	138.00 ± 3.83	0.980
K (mmol/L)	4.39 ± 0.802	4.48 ± 0.977	4.81 ± 1.05	0.303
Chloride (mmol/L)	99.00 ± 5.29	99.19 ± 4.22	97.50 ± 3.85	0.517
Bicarbonate (mmol/L)	22.65 ± 4.15	20.69 ± 3.40	21.88 ± 4.86	0.305
Calcium (mg/dl)	8.69 ± 0.486	8.81 ± 0.773	8.72 ± 0.762	0.828
Phosphorus (mg/dl)	3.43 ± 1.77	3.89 ± 1.56	4.68 ± 2.21	0.138
Magnesium (mmol/L)	1.67 ± 0.337	1.77 ± 0.267	1.79 ± 0.370	0.477
Total bilirubin (mg/dl)	0.52 ± 3.53	0.75 ± 0.589	0.54 ± 0.500	0.116
Alanine transaminase (IU/L)	39.76 ± 26.56	53.76 ± 47.98	59.44 ± 119.17	0.312
Total protein (g/dl)	6.95 ± 0.798	7.01 ± 0.747	6.61 ± 0.959	0.271
Partial thromboplastin time	46.74 ± 40.22	44.33 ± 26.72	33.42 ± 6.60	0.473

Relevant medical histories for patients in the established cardiomyopathy and new-onset cardiomyopathy groups are noted in Table 2. The etiology of the cardiomyopathy in the new-onset cardiomyopathy group was not defined since cardiovascular testing was deferred in view of the pandemic. A history of atrial fibrillation or flutter along with coronary artery disease was more commonly reported in the established cardiomyopathy group. The prevalence of stroke, chronic obstructive pulmonary disease, diabetes mellitus, systemic hypertension, hyperlipidemia, chronic kidney disease, and obstructive sleep apnea were otherwise similar between both groups. 

**Table 2:** Past medical history of patients enrolled in the established cardiomyopathy and new-onset cardiomyopathy groups. *Statistically significant. NS: non-significant, PAD: peripheral artery disease, HIV: human immunodeficiency virus, AF: atrial fibrillation, AFL: atrial flutter, COPD: chronic obstructive pulmonary disease, PE: pulmonary embolism, DVT: deep venous thrombosis, OSA: obstructive sleep apnea, CKD: chronic kidney disease, ESRD: end-stage renal disease, CAD: coronary artery disease, ACE-I: angiotensin-converting enzyme inhibitor, ARB: angiotensin receptor blocker, OAC: oral anticoagulant use.

Established CM (N = 17)	New-onset CM (N = 16)	P-value
Active cancer	1/17 (5.9%)	1/16 (6.3%)	0.9623
Asthma	1/17 (5.9%)	1/16 (6.3%)	0.9623
Hyperlipidemia	15/17 (88.2%)	10/16 (62.5%)	0.0901
Hypertension	17/17 (100%)	14/16 (87.5%)	0.1386
Diabetes mellitus	9/17 (52.9%)	9/16 (56.3%)	0.8469
Stroke	1/17 (5.9%)	1/16 (6.3%)	0.9623
PAD	2/17 (11.8%)	0/16 (0%)	NS
HIV	0	0	
Obesity	6/17 (35.3%)	8/16 (50.0%)	0.4004
AF/AFL	6/17 (37.5%)	1/16 (6.3%)	0.0343
COPD	2/17 (11.8%)	2/16 (12.5%)	0.9517
Home O_2_ use	0	0	
PE/DVT	0	2/16 (12.5%)	NS
OSA	2/17 (11.8%)	2/16 (12.5%)	0.9517
CKD-none	7/17 (41.2%)	10/16 (62.5%)	0.2282
CKD-stage 2	0	0	NS
CKD-stage 3A	1/17 (5.9%)	0	NS
CKD-stage3B	5/17 (29.4%)	0	NS
CKD-stage 4	0	0	NS
CKD-stage 5	1/17 (5.9%)	0	NS
ESRD	3/17 (17.6%)	6/16 (37.5%)	0.2064
CAD	9/17 (52.9%)	2/16 (12.5%)	0.0154*
Aspirin	12/17 (70.6%)	6/16 (37.5%)	0.0602
Plavix	4/17 (23.5%)	0	NS
OAC	4/17 (23.5%)	2/16 (12.5%)	0.4199
Statin	11/17 (64.7%)	9/16 (56.3%)	0.6269
Spironolactone use	3/17 (17.6%)	1/16 (5.9%)	0.3075
Angiotensin receptor/neprilysin inhibitor	1/17 (5.9%)	0	NS
Diuretic	11/17 (64.7%)	4/16 (25.0%)	0.0242*
Beta-blocker	16/17 (94.1%)	8/16 (50.0%)	0.0051*
ACE-I/ARB	10/17 (58.8%)	9/16 (56.3%)	0.8863

The results of the primary endpoint of the study are found in Table 3. No difference was noted in the mortality rate or admission to the medical intensive care unit between all three groups. However, the length of stay in the established cardiomyopathy group (6.88 days) was significantly lower than the length of stay of the patients in the control group (11.83 days) and in the new-onset cardiomyopathy group (13.25 days), with a P-value of 0.017. Also, significantly more patients in the control group required non-invasive and/or mechanical ventilation upon admission to our hospital system (p-value of 0.001).

**Table 3:** Primary endpoints (average values). *Statistically significant. Values were expressed as mean (±SD), whereas for categorical variables, values were expressed as n (%). LDH: lactate dehydrogenase.

	Control (N = 78)	Established CM (N = 17)	New-onset CM (N = 16)	P-value
Mortality rate	20 (25.6%)	7 (41.2%)	7 (43.75%)	0.1985
Admission to ICU	23 (29.5%)	2 (11.8%)	6 (37.5%)	0.0901
Length of stay	11.83 ± 6.85	6.88 ± 6.50	13.25 ± 8.27	0.017*
Disposition at discharge				0.332
Home	42 (53.8%)	8 (47.06%)	8 (50.0%)	
Facility	16 (20.5%)	2 (11.76%)	1 (6.3%)	
Died	17 (21.8%)	7 (41.2%)	7 (43.75%)	
Hospice	3 (3.8%)	0	0	
Oxygenation status				
Room air	11 (14.1%)	7 (41.18%)	5 (31.25%)	0.001*
Non-invasive ventilation	20 (25.6%)	9 (52.94%)	4 (25.0%)	
Mechanical ventilation	47 (60.3%)	1 (5.88%)	7 (43.75%)	
Cardiac biomarkers				
Troponin T (ng/ml)	0.07 ± 0.13	0.09 ± 0.123	0.11 ± 0.284	0.619
Creatinine kinase (IU/L)	542.21 ± 987.23	483.30 ± 477.904	280.08 ± 222.41	0.638
Pro-BNP (pg/ml)	2018.55 ± 5256.23	14404.54 ± 15297.99	8788.8 ± 14094.33	0.001*
Markers of acute illness				
White blood cell count (1000/µL)	7.71 ± 4.99	8.08 ± 3.06	8.82 ± 3.55	0.678
Erythrocyte sedimentation rate (mm/HR)	69.55 ± 29.59	96.0 ± 24.04	82.5 ± 38.89	0.502
C-reactive protein (mg/L)	126.21 ± 86.62	111.34 ± 69.41	119.41 ± 83.27	0.819
Procalcitonin (ng/ml)	1.93 ± 5.96	2.93 ± 4.75	1.12 ± 1.43	0.677
Ferritin (ng/ml)	2304.74 ± 3535.71	2239.75 ± 2531.12	4075.81 ± 6256.08	0.271
D-dimer (ng/ml)	1726.8 ± 1604.2	647.52 ± 525.69	2972.00 ± 2257.89	0.114
LDH (IU/L)	512.01 ± 236.53	553.36 ± 278.98	481.07 ± 290.36	0.774
Lactate (mmol/L)	1.71 ± 0.979	2.64 ± 1.64	3.70 ± 4.14	0.011*
Albumin (mg/dl)	3.45 ± 0.442	3.51 ± 0.381	3.32 ± 0.526	0.437
Fibrinogen	494.1 ± 122.557	484.5 ± 238.295	762.67 ± 124.917	0.031*
Alanine aminotransferase	72 ± 28.821	107.59 ± 85.244	71.84 ± 30.25	0.008*
Aspartate transaminase	60.75 ± 27.251	126.76 ± 154.51	69.94 ± 51.55	0.002*
QTc interval	442.35 ± 41.797	494.38 ± 29.7	472.21 ± 42.26	0.001*
Ejection fraction	60.64% ± 5.24%	29.18% ± 12.02%	31.25% ± 8.85%	0.001*

Disposition at discharge and troponin T levels on admission were not significantly different across all three groups. The pro-BNP level in the established cardiomyopathy group was significantly higher compared to the control group but no different than that of the new-onset cardiomyopathy group.

The white blood cell count, erythrocyte sedimentation rate, C-reactive protein, pro-calcitonin, ferritin, D-dimer, lactate dehydrogenase, and albumin levels were all similar between the three groups. The lactate level was significantly different in the established cardiomyopathy group compared to the control group but no different from the new-onset cardiomyopathy group. The fibrinogen level in the new-onset cardiomyopathy group was significantly higher than that of the established cardiomyopathy and control groups.

A subgroup analysis (Table 4) based on ejection fraction cutoffs (mildly reduced ejection fraction, moderately reduced ejection fraction, and severely reduced ejection fraction) failed to reveal a significant difference in the established cardiomyopathy and new-onset cardiomyopathy groups with regards to mortality rate and admission to the medical intensive care unit. However, the length of stay in patients with severely reduced ejection fraction was significantly longer in the new-onset cardiomyopathy group versus the established cardiomyopathy group (15.38 vs 5.78 days; P = 0.011). 

**Table 4:** Sub-group analysis (average values). *Statistically significant. ns: non-significant, EF: ejection fraction.

Established CM (N = 17)	New-onset CM (N = 16)	P-value
Mortality rate			
Mildly reduced EF (≥40%-<50%)	2/5 (40.0%)	3/4 (75.0%)	0.0457*
Moderately reduced EF (≥30%-<40%)	0/7	0/7	NS
Severely reduced EF (<30%)	5/9 (55.6%)	4/8 (50.0)	0.7511
Admission to ICU			
Mildly reduced EF (≥40%-<50%)	0/5	2/4 (50%)	NS
Moderately reduced EF (≥30%-<40%)	1/3 (33%)	1/4 (25%)	0.6186
Severely reduced EF (<30%)	1/9 (11.1%)	3/8 (37.5%)	0.0801
Length of stay			
Mildly reduced EF (≥40%-<50%)	8.4 ± 7.63	12.25 ± 6.65	0.413
Moderately reduced EF (≥30%-<40%)	7.67 ± 8.32	10.0 ± 4.97	0.400
Severely reduced EF (<30%)	5.78 ± 5.911	15.38 ± 10.25	0.011*

## Discussion

In this retrospective study involving patients admitted with a primary diagnosis of viral pneumonia secondary to COVID-19 between March 1 and April 30, 2020, there was no difference in the mortality rate, admission rate to the medical intensive care unit, troponin level on admission and disposition at discharge between patients with normal left ventricular systolic function (control), established cardiomyopathy and new-onset cardiomyopathy groups. Although there was no difference in the length of stay between the control group and the new-onset cardiomyopathy group, the length of stay in the established cardiomyopathy group was markedly lower than that of the control and new-onset cardiomyopathy groups. Four of the sixteen patients in the established cardiomyopathy group either had a do not resuscitate (DNR) or do not intubate (DNI) order on admission or by discharge, whereas the patients in the new-onset cardiomyopathy and the control groups were all full code. Furthermore, another two of the six patients that died in the established cardiomyopathy group had limited time for cardiopulmonary resuscitation (<15 minutes) since care was deemed futile. We, therefore, suspect that there were more discussions about palliative care involvement and end-of-life care in this group, which could explain the reduced length of stay, although this remains speculative and was never confirmed in our study.

Most of the laboratory parameters documented on admission were not significantly different between all three groups, and those included troponin T, white blood cell count, erythrocyte sedimentation rate, C-reactive protein, procalcitonin, ferritin, D-dimer, lactic dehydrogenase, and albumin. It appears that the lactate and fibrinogen levels were the highest in the new-onset cardiomyopathy group (P = 0.011 and P = 0.031, respectively). The discrepancy of the laboratory findings couldn’t be explained; nevertheless, they didn’t appear to contribute to certain primary endpoints (i.e., mortality rate and admission rate to the intensive care unit). Most patients in the control group either required non-invasive or invasive ventilation on admission, and that was significantly different than the other two groups (P-value = 0.001), and we suspect that that early COVID-19 experience included a high proportion of patients that were admitted with marked hypoxemia followed by profound respiratory failure resulting in endotracheal intubation, which is unlikely to be the case today with the higher prevalence of more infectious strains accompanied by milder symptoms and the subsequent development of various vaccines [7]. 

The current coronavirus pandemic has certainly taken a toll on our society. A retrospective study evaluating the mortality rate of patients admitted with COVID-19, including 42,604 patients hospitalized between March 1 and November 2020, revealed a mortality rate of around 12.5% for men and 9.6% for women [8]. Another study reported a mortality rate of 31% in patients whose hospitalization course included acute respiratory distress syndrome, which is similar to what we found in our study [9]. Additionally, African Americans were at greater risk of being hospitalized for complications from COVID-19 and had a higher mortality rate compared to other races, particularly Caucasians [9]. 

COVID-19 should be regarded as a systemic illness based on its multiorgan system involvement, which includes but is not limited to a hypercoagulable state, arrhythmias, myocarditis, acute kidney injury, and the development of various psychiatric disorders such as anxiety and depression [9]. Although the respiratory tract and lungs are the most involved in COVID-19, with acute respiratory distress syndrome being the most feared complication, significant cardiac morbidity has been reported in the form of elevated cardiac biomarkers [10]. Mechanisms include direct viral infection of myocytes, myocardial injury secondary to the immune response initiated against the virus, microvascular ischemia and coronary embolism from a hypercoagulable state [10]. The acute coronary syndrome has also been shown to be more prevalent in patients with COVID-19, and that is likely due to the overall proinflammatory state, as IL-6 has been shown to promote vascular inflammation [9]. The ACE2 receptor, expressed in the lungs and heart, has been implicated as a possible target of the virus, hence the increased risk of acute respiratory distress syndrome and myocarditis [9]. The CDC reported that the risk of myocarditis is 16 times more in patients hospitalized with COVID-19 compared to the rest of the population and that vaccination significantly reduces the risk of myocarditis and other cardiac complications [5]. The incidence of myocarditis reported by the CDC was around 150 cases per 100,000 patients with COVID-19 [5]. Whereas, in another study, the risk of myocarditis following the Pfizer COVID-19 vaccination was around 2.13 cases per 100,000, therefore making a compelling argument for continued vaccination [11]. 

Our study was not designed to establish causality, as very little cardiovascular testing was performed to limit the spread of the virus. Nevertheless, we were able to obtain limited echocardiograms if there was an appropriate indication. Although our prediction was that patients with established cardiomyopathy and new-onset cardiomyopathy had more comorbidities and were likely sicker, it appears that the number of patients that required endotracheal intubation on admission in the control group was higher than anticipated, which resulted in an overall higher mortality rate than what was expected. This may have led to there not being a significant difference with regards to mortality between all three groups.

Our study had multiple limitations that were unavoidable. Foremost, this was a retrospective analysis with inherent limitations. Although we had more than 1163 patients admitted to our hospital system with a primary diagnosis of COVID-19 between March 1, 2020, and April 30, 2020, only a small proportion of those patients were enrolled in the study since having a limited echocardiogram demonstrating normal left ventricular systolic function (ejection fraction greater or equal to 50%) was a requirement during the hospital stay for enrollment into the control group. Second, not all races were represented in our dataset, and we, therefore, had to include one race in our final analysis. Third, our definition of new-onset cardiomyopathy included no prior history of heart failure with a limited echocardiogram demonstrating a left ventricular ejection fraction of less than 50%. Fourth, patients who had an established history of cardiomyopathy were enrolled in our study with or without an echocardiogram demonstrating the stability of left ventricular systolic impairment. Extensive cardiovascular testing was not performed, and therefore the true incidence of COVID-19-induced cardiomyopathy during the study period was not defined.

## Conclusions

Our center’s 2020 COVID-19 experience suggests that there was no significant difference in mortality rate and admission rate to the intensive care unit between patients with normal left ventricular systolic function, established cardiomyopathy and new-onset cardiomyopathy and that was likely driven by the high incidence of progressive respiratory failure. The length of stay between the group of patients with normal left ventricular systolic function and the new-onset cardiomyopathy group was similar but different from the established cardiomyopathy group and that was likely explained by more discussions about palliative care involvement and end-of-life care. Further research is required to elucidate the true incidence of cardiac involvement secondary to COVID-19 and its repercussions.
